# Supplementary material for: A topographic atlas defines developmental origins of cell heterogeneity in the human embryonic lung
Source: Nat Cell Biol. 2023 Jan 16;25(2):351–65. doi: 10.1038/s41556-022-01064-x (PMC9928586; doi:10.1038/s41556-022-01064-x)
Supplement: Supplementary file 1 — Supplementary Note 1. [file 41556_2022_1064_MOESM1_ESM.pdf]

nature cell biology

Resource

https://doi.org/10.1038/s41556-022-01064-x

A topographic atlas defines developmental origins of cell heterogeneity in the human embryonic lung

In the format provided by the authors and unedited

Supplementary Note 1

of the: “A topographic atlas defines developmental origins of cell heterogeneity in the human embryonic lung”

Immune cells

Immune cells colonize the lung during the first trimester of gestation, showing signs of functional competence

In the first trimester of gestation, liver is the main source of immune cells that seed various organs, including lung, to produce their resident immune cells[1]. Re-clustering of 4113 PTPRC^{pos}[2] immune cells and megakaryocytes suggested 22 clusters, which were annotated to distinct immune cell-types and their corresponding immature states (Suppl. Note Fig. 1A-B, Suppl. Table 1-10). In summary, embryonic lungs contain myeloid macrophages, monocytes, neutrophils, mast/basophis, dendritic cells (DCs), lymphoid B-cells, innate lymphoid cells (ILCs) natural killer (NK) cells and megakaryocytes. Myeloid cells were more abundant in early stages of development whereas lymphoid cells appeared after 10 pcw (Suppl. Note Fig. 1C-D).

Macrophages presented the most numerous immune cell-type, including two immature (-0 and -22) and one mature cluster (cl-16), that expressed high *TNF* levels, suggesting that it represents functionally competent macrophages[3]. Examination of genes encoding known myeloid-cell effector proteins, such as the cytokines *IL1A*, IL1B[4], *IL23A*[5] and the chemokines *CCL4* and *CXCL2*[6] provided additional evidence that both macrophages and monocytes (immune cl-14) can be operative at this early stage (Suppl. Note Fig. 1B, E). The myeloid lineage also included: (i) the *MPO*^{pos} [7] neutrophils, with a small percentage of them being *ELANE*^{pos} [8], suggesting that they are still immature (Suppl. Note Fig. 1B), (ii) *MKI67*^{pos} *PCNA*^{pos} proliferating myeloid cells (cl-8) (Suppl. Note Fig. 1B) and (iii) three types of dendritic cells expressing *HLA-DQA1*[9]. Among these, only cl-5 expressed *CCR7*, suggesting that it contains migrating dendritic cells[10] and only cl-19 expressed *CLEC9A*, a conventional dendritic cell marker[11].

The lymphoid lineage encompassed five distinct clusters (Suppl. Note Fig. 1A). The most abundant cell-type (cl-1) corresponded to *NKG7*^{pos} *NCR1*^{pos} *KLRD1*^{pos} NK-cells (Suppl. Note Fig. 1B). They expressed genes encoding the known effector proteins *IFNG*[12] and *GNLY* and the granzymes A and M[13] indicating that NK-cells are functional at this stage (Suppl. Note Fig. 1F). cl-13 contained *GATA3*^{pos} *IL1RL1*^{pos} innate lymphoid cells 2 (ILC2) and cl-21 *IL1R1*^{pos}

*IL23R*pos ILC3 cells. While both clusters were *KIT*pos[14] (Add. Fig. 1B), only ILC2 expressed *IL13*, as previously reported[15] (arrow in Suppl. Note Fig. 1F).

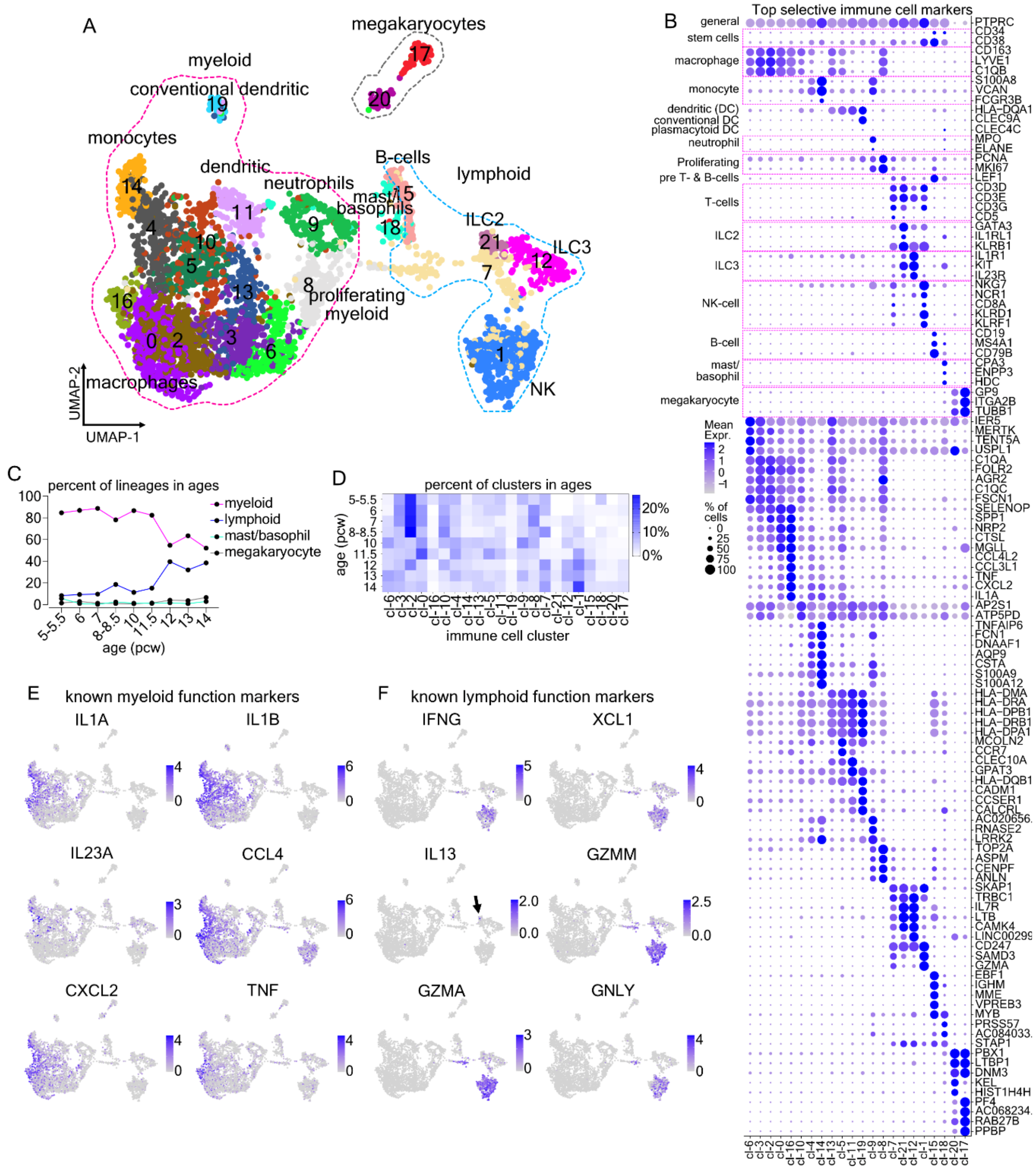

Supplementary Note Figure 1. Immune cell characterization suggests thar all main cell-types are found in the embryonic lung, being functionally competent. (A) UMAP-plot of the 4113 analyzed immune cells. Different colors indicate the 22 suggested clusters. Dotted outlines show the myeloid (magenta), lymphoid (cyan) lineages and megakaryocytes (gray). **(B)** Balloon-plot of known immune cell marker genes (*PTPRC-TUBB1*). General: *PTPRC*[2], Stem cells: *CD34, CD38*[1], Macrophages: *CD163, LYVE1*[16], *C1QB*[9], Monocytes: *S100A8*, *VCAN*[9], *FCGR3B*[17], Dendritic cells: *HLA-DQA1* (general), *CLEC4C* (plasmacytoid)[9], *CLEC9A* (myeloid)[18], Neutrophils: *MPO*, *ELANE*[1], Proliferating: *MKI67*[19], *PCNA*[20], T-cells: *CD3D*, *CD3E*, *CD3G*[9, 21], *CD5*[22], ILC2: *GATA3*, *IL1RL1*[23], *KLRB1* (CD161)[24], ILC3: *IL1R*, *IL23R* , *KIT*[25], NK-cells: *NKG7*, *NCR1*, *KLRD1*, *KLRF1*[9], *CD8A*[1], B-cells: *CD19*, *MS4A1*, *CD79B*[9], Mast/basophils: *CPA3*,

HDC[26], *ENPP3*[27] and *GP9*[28], *ITGA2B*[9], *TUBB1*[29]. The remaining genes correspond to the top-5, most selective genes for each cluster. From the statistically significant genes (adjusted p-value < 0.001, according to MAST (Bonferroni correction using all features)), the top-10 genes (based on average log2 fold-change) were sorted according to the percent of positive cells and the top-5 markers were plotted. Gene order follows the cluster order. Balloon size: percent of positive cells. Color intensity: scaled expression. Blue: high, Gray: low. **(C)** Line plot of lineage abundancies (%) in the analyzed ages. **(D)** Heatmap of cluster abundancies (%) in the analyzed ages. Dark blue: high, White: zero. **(E-F)** UMAP-plots of gene expression levels of known myeloid (E) and lymphoid (F) function genes. Expression levels correspond to log_2(normalized UMI-counts+1) (library size was normalized to 10.000). Blue: high, Gray: zero.

The lymphoid cell cl-7 was positive for the general T-cell marker genes *CD3D*, *CD3G*, *CD3G*[21] and presumably corresponds to immature lymphoid cells, while cl-15 contained *CD19*^{pos} *CD79B*^{pos} *MS4A1*^{pos} B-cells (Suppl. Note Fig. 1B). cl-18 cells expressed basophil and mast cell markers like *CPA3*, *HDC*[26] and *ENPP3*[27] but the low percent of positive cells in the cluster suggests that they are still immature (Suppl. Note Fig. 1B). Finally, we detected two populations of *GP9*^{pos} [28] megakaryocytes, which might correspond to immature (cl-20) and mature (cl-17) cell-states, based on the higher expression levels of differentiation markers like *GP9*, *ITGA2B* and *TUBB1* in cl-17 (Suppl. Note Fig. 1B).

Our experiments cannot assess whether the lung immune cells during the first trimester derive from the circulation or represent lung resident cells like alveolar macrophages[30] and ILCs[23].We have confirmed that the major immune cell-types are detected in the developing lung, earlier than previously reported[31, 32] and that they express distinct effector genes, suggesting that they are functional.

Endothelial cells

The main endothelial cell-types appear during the early stages of lung development

The separate analysis of the endothelial cell group indicated the presence of arterial (*CXCL12, IGFBP3, GJA5, DKK2*), venous (*CPE, CDH11*), capillary (*SGK1*, *NTRK2*), lymphatic (*PROX1*, *CCL21*) and bronchial (*HBEGF*) endothelial clusters, which were annotated according to the expression of known markers of the adult human lung [9, 33] (Suppl. Note Fig. 2A-B, Suppl. Table 1-11). Three of the clusters (-3, -10 and -14) included proliferating cells, as indicated *MKI67*[19], *PCNA* [20] expression (Suppl. Note Fig. 2A-B). The remaining clusters were annotated as immature cell-states, expressing lower levels of differentiation markers and higher *KIT*, as previously described[34]. Both mature and immature endothelial clusters were present during all analyzed stages suggesting that lung angiogenesis is asynchronous, at least during the first trimester (Suppl. Note Fig. 2C).

The developmental trajectory of arterial endothelium

The arterial trajectory of cl-12, -6 and -9 was the most prominent one among the endothelial cells (Suppl. Note Fig. 2A). The pseudotime analysis of these clusters together with the immature cl-0 and -1 confirmed the ordering of the cells, which was also supported by the scVelo arrow directions and the elevated differentiation markers in mature cells (Suppl. Note Fig. 2D-F). We defined five statistically significant gene modules changing along the trajectory (Suppl. Note Fig. 2F). Module-1 contained immature cell (cl-0, -1) markers, such as the capillary marker *CA4*[33] and the adult aerocyte marker *HPGD*[33, 35], which, might control endothelial cell migration and vessel formation[36] by inactivating prostaglandins, as previously reported[37]. Immature cells expressed *LHX6*, encoding a TF involved in neuronal differentiation [33, 35, 38], which was downregulated in the mature arterial clusters. Module-2 included upregulated genes in the immature cl-12 and -6, such as the *INSR*, that controls endothelial cell sprouting down-stream of VEGF-signaling[39]. The other three modules contained genes that are progressively activated in mature cells.

The BMP-pathway mediator *SMAD1* was enriched in the immature cl-12, in agreement with its role in promoting the expression of NOTCH-signaling components (e.g., *HES4*, *JAG1* and *HEY1*) (Suppl. Note Fig. 2G), which in turn, regulate the tip- versus stalk- cell selection in the developing arterial endothelium[40]. More mature arterial cells expressed the inhibitory *SMAD6*[41], which downregulates cell proliferation and induces genes encoding cell junction components[42], downstream of NOTCH-signaling. These results suggest an interplay between BMP and NOTCH pathways during arterial differentiation in the human lung.

The transient activation of the collagen genes, *COL15A1*[33] and *COL18A1* in immature arterial cells and the up-regulation of the elastogenesis-related genes, *FBLN5* and *LTBP4*[43] in the most mature cl-9 (Suppl. Note Fig. 2F, H) suggested that arterial endothelial cells at different maturation states produce different ECM proteins and contribute to arterial wall formation.

Arterial endothelium communicates with pericytes

Spatial co-localization analysis showed that within the vessel neighborhood (Fig. 1C), there is high spatial correlation between endothelium and pericytes involving only mature arterial endothelial cells (cl-9) (Suppl. Note Fig. 2I). This indicates that pericytes cover the vessels only when endothelial cells reach an advanced maturation state. The interactome analysis between the involved mesenchymal and endothelial clusters confirmed known communication-patterns like the PDGF and EDN pathways that induce pericyte migration and

proliferation[44]. In particular, *PDGFB* was detected in all analyzed endothelial cells and it mainly signals through PDGFRB to pericytes (Suppl. Note Fig. 2J). *EDN1* was highly expressed by immature endothelial cl-0 and -2 (Suppl. Note Fig. 2K). On the other hand, *HGF* was the only predicted ligand to be exclusively expressed by pericytes and target the arterial endothelial cells (cl-6, -9), possibly contributing to endothelial cell growth and tube formation, as in the rat lung[45] (Add. Suppl. Note Fig. 2L). Lastly, *DHH* was detected only in the mature arterial endothelium, targeting the mesenchyme, in agreement with its known role in angiogenesis[46] (Suppl. Note Fig. 2M).

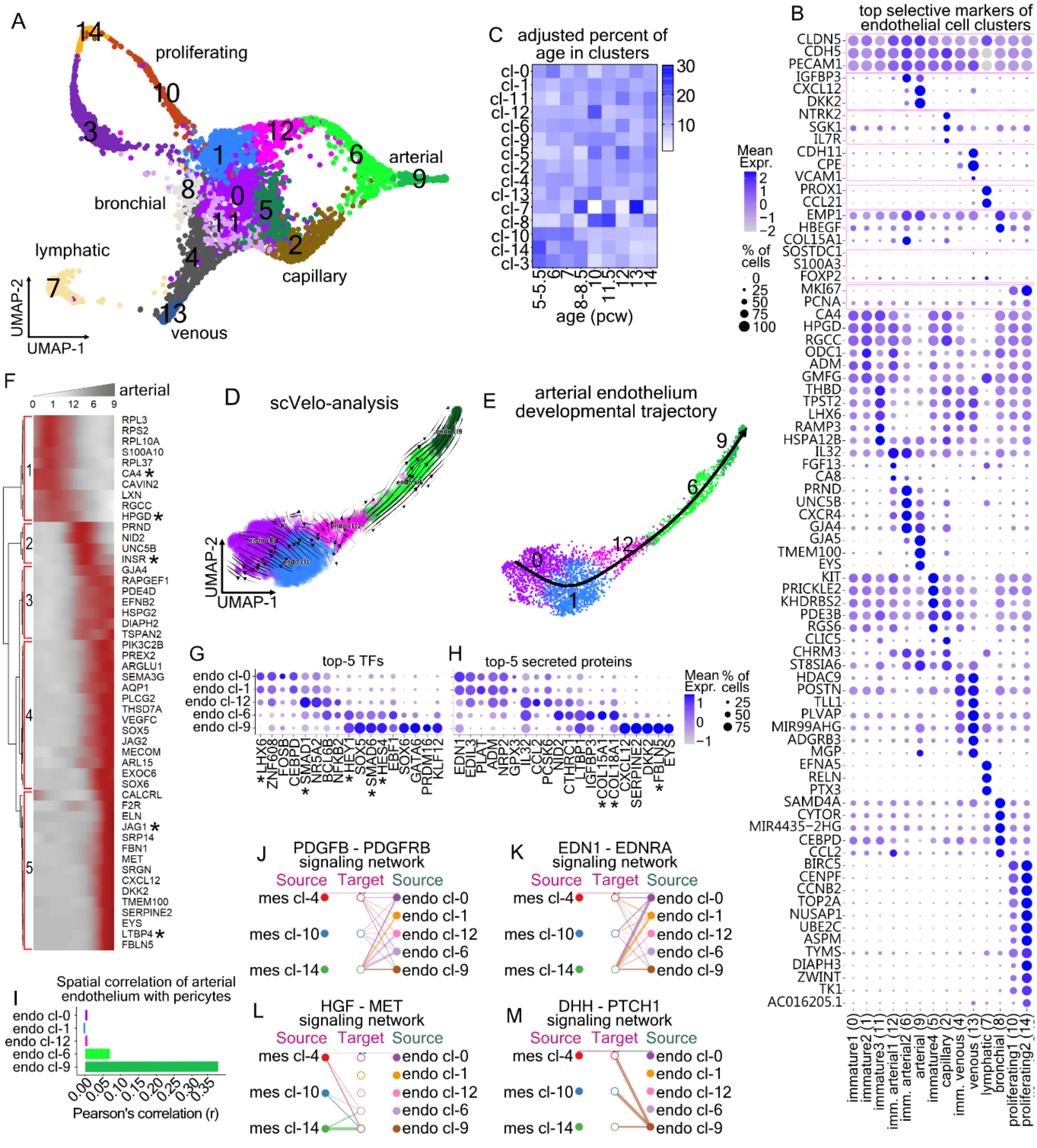

Supplementary Note Figure 2. Analysis of endothelial cell heterogeneity. (A) UMAP-plot of the 6629 endothelial cells. Different colors indicate the 15 suggested clusters. **(B)** Balloon plot

of known endothelial cell marker genes (*CLDN5-PCNA*). General: *CLDN5*, *PECAM1*[9], *CDH5*[33], Arterial: *IGFBP3*, *CXCL12*, *DKK2*[9], Capillary: *NTRK2*, *SGK1*[9], *IL7R*[33], Venous: *CDH11*, *CPE*, *VCAM1*[33], Lymphatic: *PROX1*[47, 48], *CCL21*[9], Bronchial: *EMP1*, *HBEGF*[9], *COL15A1*[33], Aerocytes: *SOSTDC1*, *S100A3*[9], *FOXP2*[33] and Proliferating: *MKI67*[19], *PCNA*[20]. The remaining genes correspond to the top-5, most selective genes for each cluster. *COL15A1*[33] was not expressed in cl-8 but in immature arterial cl-6, suggesting differences between embryonic and adult lung endothelium. **(C)** Heatmap illustrating the relative abundance of endothelial clusters at different developmental stages. To avoid bias, cell numbers were firstly normalized according to dataset size per stage. Dark blue: high, White: zero. **(D)** scVelo-analysis on the neuronal cells. Colors as in "A" and direction of arrows shows progression towards more differentiated stages. **(E)** Pseudotime analysis of the arterial endothelial trajectory, with Slingshot. **(F)** Heatmap of the top-50 differentially expressed genes along the arterial trajectory, according to tradeSeq. The numbers on the left correspond to 5 stable gene-modules (red squares) (Bootstrap values module-1: 0.99, module-2: 0.89, module: 0.79, module-4: 0.83, module-5: 0.90). "*" indicate commended genes. Color intensity: scaled expression. Dark red: high, Gray: low. **(G-H)** Top-5 secreted proteins (G) and transcription factors (H), in the clusters of the trajectory. **(I)** Bar-plot of Pearson's correlation between pericytes (mes cl-14) and the suggested endothelial clusters of the arterial trajectory, based on ST-data. Colors as in "A". **(J-M)** CellChat hierarchical plots of ligand-receptor pairs for *PDGFB* (J), *EDN1* (K), *HGF* (L) and *DHH* (M). In all Balloon plots, from the statistically significant genes (adjusted p-value < 0.001, according to MAST (Bonferroni correction using all features)), the top-10 genes (based on average log2 fold-change) were sorted according to the percent of positive cells and the top-5 markers were plotted. Gene order follows the cluster order. Balloon size: percent of positive cells. Color intensity: scaled expression. Blue: high, Gray: low.

References

1. Popescu, D.M. *et al.* Decoding human fetal liver haematopoiesis. *Nature* **574**, 365-371 (2019).
2. Hermiston, M.L., Xu, Z. & Weiss, A. CD45: a critical regulator of signaling thresholds in immune cells. *Annu Rev Immunol* **21**, 107-137 (2003).
3. Parameswaran, N. & Patial, S. Tumor necrosis factor-alpha signaling in macrophages. *Crit Rev Eukaryot Gene Expr* **20**, 87-103 (2010).
4. Di Paolo, N.C. & Shayakhmetov, D.M. Interleukin 1alpha and the inflammatory process. *Nat Immunol* **17**, 906-913 (2016).
5. Pirhonen, J., Matikainen, S. & Julkunen, I. Regulation of virus-induced IL-12 and IL-23 expression in human macrophages. *J Immunol* **169**, 5673-5678 (2002).
6. Mantovani, A. *et al.* The chemokine system in diverse forms of macrophage activation and polarization. *Trends Immunol* **25**, 677-686 (2004).
7. Klebanoff, S.J., Kettle, A.J., Rosen, H., Winterbourn, C.C. & Nauseef, W.M. Myeloperoxidase: a front-line defender against phagocytosed microorganisms. *J Leukoc Biol* **93**, 185-198 (2013).
8. Takahashi, H. *et al.* Structure of the human neutrophil elastase gene. *J Biol Chem* **263**, 14739-14747 (1988).
9. Travaglini, K.J. *et al.* A molecular cell atlas of the human lung from single-cell RNA sequencing. *Nature* **587**, 619-625 (2020).
10. Riol-Blanco, L. *et al.* The chemokine receptor CCR7 activates in dendritic cells two signaling modules that independently regulate chemotaxis and migratory speed. *J Immunol* **174**, 4070-4080 (2005).

11. Yamasaki, S., Edn. 1st 1 online resource (VI, 215 pages 229 illustrations, 216 illustrations in color (Springer Japan : Imprint: Springer,, Tokyo; 2016).
12. Guo, Y., Patil, N.K., Luan, L., Bohannon, J.K. & Sherwood, E.R. The biology of natural killer cells during sepsis. *Immunology* **153**, 190-202 (2018).
13. Dotiwala, F. & Lieberman, J. Granulysin: killer lymphocyte safeguard against microbes. *Curr Opin Immunol* **60**, 19-29 (2019).
14. Meininger, I. *et al.* Tissue-Specific Features of Innate Lymphoid Cells. *Trends Immunol* **41**, 902-917 (2020).
15. Nagasawa, M. *et al.* KLRG1 and NKp46 discriminate subpopulations of human CD117(+)CRTH2(-) ILCs biased toward ILC2 or ILC3. *J Exp Med* **216**, 1762-1776 (2019).
16. Chakarov, S. *et al.* Two distinct interstitial macrophage populations coexist across tissues in specific subtissular niches. *Science* **363** (2019).
17. Bertrand, G., Duprat, E., Lefranc, M.P., Marti, J. & Coste, J. Characterization of human FCGR3B*02 (HNA-1b, NA2) cDNAs and IMGT standardized description of FCGR3B alleles. *Tissue Antigens* **64**, 119-131 (2004).
18. Canton, J. *et al.* The receptor DNGR-1 signals for phagosomal rupture to promote cross-presentation of dead-cell-associated antigens. *Nat Immunol* **22**, 140-153 (2021).
19. Schonk, D.M. *et al.* Assignment of the gene(s) involved in the expression of the proliferation-related Ki-67 antigen to human chromosome 10. *Hum Genet* **83**, 297-299 (1989).
20. Bologna-Molina, R., Mosqueda-Taylor, A., Molina-Frechero, N., Mori-Estevez, A.D. & Sanchez-Acuna, G. Comparison of the value of PCNA and Ki-67 as markers of cell proliferation in ameloblastic tumors. *Med Oral Patol Oral Cir Bucal* **18**, e174-179 (2013).
21. Dong *et al.* Structural basis of assembly of the human T cell receptor-CD3 complex. *Nature* **573**, 546-552 (2019).
22. Domingues, R.G. *et al.* CD5 expression is regulated during human T-cell activation by alternative polyadenylation, PTBP1, and miR-204. *Eur J Immunol* **46**, 1490-1503 (2016).
23. Dahlgren, M.W. *et al.* Adventitial Stromal Cells Define Group 2 Innate Lymphoid Cell Tissue Niches. *Immunity* **50**, 707-722 e706 (2019).
24. Bal, S.M., Golebski, K. & Spits, H. Plasticity of innate lymphoid cell subsets. *Nat Rev Immunol* **20**, 552-565 (2020).
25. Spits, H. *et al.* Innate lymphoid cells--a proposal for uniform nomenclature. *Nat Rev Immunol* **13**, 145-149 (2013).
26. Dwyer, D.F., Barrett, N.A., Austen, K.F. & Immunological Genome Project, C. Expression profiling of constitutive mast cells reveals a unique identity within the immune system. *Nat Immunol* **17**, 878-887 (2016).
27. Buhring, H.J., Streble, A. & Valent, P. The basophil-specific ectoenzyme E-NPP3 (CD203c) as a marker for cell activation and allergy diagnosis. *Int Arch Allergy Immunol* **133**, 317-329 (2004).
28. Bianchi, E., Norfo, R., Pennucci, V., Zini, R. & Manfredini, R. Genomic landscape of megakaryopoiesis and platelet function defects. *Blood* **127**, 1249-1259 (2016).
29. Freson, K. *et al.* The TUBB1 Q43P functional polymorphism reduces the risk of cardiovascular disease in men by modulating platelet function and structure. *Blood* **106**, 2356-2362 (2005).
30. Hussell, T. & Bell, T.J. Alveolar macrophages: plasticity in a tissue-specific context. *Nat Rev Immunol* **14**, 81-93 (2014).
31. de Kleer, I.M. *et al.* Perinatal Activation of the Interleukin-33 Pathway Promotes Type 2 Immunity in the Developing Lung. *Immunity* **45**, 1285-1298 (2016).
32. Michaelsson, J., Mold, J.E., McCune, J.M. & Nixon, D.F. Regulation of T cell responses in the developing human fetus. *J Immunol* **176**, 5741-5748 (2006).
33. Schupp, J.C. *et al.* Integrated Single Cell Atlas of Endothelial Cells of the Human Lung. *Circulation* (2021).

34. Suzuki, T. *et al.* c-Kit immunoexpression delineates a putative endothelial progenitor cell population in developing human lungs. *Am J Physiol Lung Cell Mol Physiol* **306**, L855-865 (2014).
35. Gillich, A. *et al.* Capillary cell-type specialization in the alveolus. *Nature* **586**, 785-789 (2020).
36. Finetti, F. *et al.* Prostaglandin E2 regulates angiogenesis via activation of fibroblast growth factor receptor-1. *J Biol Chem* **283**, 2139-2146 (2008).
37. Cho, H. *et al.* Role of glutamine 148 of human 15-hydroxyprostaglandin dehydrogenase in catalytic oxidation of prostaglandin E2. *Bioorg Med Chem* **14**, 6486-6491 (2006).
38. Zhou, C. *et al.* Lhx6 and Lhx8: cell fate regulators and beyond. *FASEB J* **29**, 4083-4091 (2015).
39. Walker, A.M.N. *et al.* Endothelial Insulin Receptors Promote VEGF-A Signaling via ERK1/2 and Sprouting Angiogenesis. *Endocrinology* **162** (2021).
40. Moya, I.M. *et al.* Stalk cell phenotype depends on integration of Notch and Smad1/5 signaling cascades. *Dev Cell* **22**, 501-514 (2012).
41. Imamura, T. *et al.* Smad6 inhibits signalling by the TGF-beta superfamily. *Nature* **389**, 622-626 (1997).
42. Ruter, D.L. *et al.* SMAD6 transduces endothelial cell flow responses required for blood vessel homeostasis. *Angiogenesis* **24**, 387-398 (2021).
43. Kumra, H. *et al.* Fibulin-4 exerts a dual role in LTBP-4L-mediated matrix assembly and function. *Proc Natl Acad Sci U S A* **116**, 20428-20437 (2019).
44. Kemp, S.S., Aguera, K.N., Cha, B. & Davis, G.E. Defining Endothelial Cell-Derived Factors That Promote Pericyte Recruitment and Capillary Network Assembly. *Arterioscler Thromb Vasc Biol* **40**, 2632-2648 (2020).
45. Seedorf, G. *et al.* Hepatocyte growth factor as a downstream mediator of vascular endothelial growth factor-dependent preservation of growth in the developing lung. *Am J Physiol Lung Cell Mol Physiol* **310**, L1098-1110 (2016).
46. Hollier, P.L. *et al.* Full-length Dhh and N-terminal Shh act as competitive antagonists to regulate angiogenesis and vascular permeability. *Cardiovasc Res* **117**, 2489-2501 (2021).
47. Wigle, J.T. *et al.* An essential role for Prox1 in the induction of the lymphatic endothelial cell phenotype. *EMBO J* **21**, 1505-1513 (2002).
48. Wigle, J.T. & Oliver, G. Prox1 function is required for the development of the murine lymphatic system. *Cell* **98**, 769-778 (1999).
