# Supplementary material for: A topographic atlas defines developmental origins of cell heterogeneity in the human embryonic lung
Source: Nat Cell Biol. 2023 Jan 16;25(2):351–65. doi: 10.1038/s41556-022-01064-x (PMC9928586; doi:10.1038/s41556-022-01064-x)
Supplement: Supplementary file 2 — Reporting Summary [file 41556_2022_1064_MOESM2_ESM.pdf]

nature portfolio

Corresponding author(s): Christos Samakovlis
Mats Nilsson

Last updated by author(s): Oct 6, 2022

Reporting Summary

Nature Portfolio wishes to improve the reproducibility of the work that we publish. This form provides structure for consistency and transparency in reporting. For further information on Nature Portfolio policies, see our Editorial Policies and the Editorial Policy Checklist.

Statistics

For all statistical analyses, confirm that the following items are present in the figure legend, table legend, main text, or Methods section.

n/a	Confirmed	
☐	☒	The exact sample size (*n*) for each experimental group/condition, given as a discrete number and unit of measurement
☒	☐	A statement on whether measurements were taken from distinct samples or whether the same sample was measured repeatedly
☐	☒	The statistical test(s) used AND whether they are one- or two-sided<br>*Only common tests should be described solely by name; describe more complex techniques in the Methods section.*
☐	☒	A description of all covariates tested
☐	☒	A description of any assumptions or corrections, such as tests of normality and adjustment for multiple comparisons
☐	☒	A full description of the statistical parameters including central tendency (e.g. means) or other basic estimates (e.g. regression coefficient) AND variation (e.g. standard deviation) or associated estimates of uncertainty (e.g. confidence intervals)
☐	☒	For null hypothesis testing, the test statistic (e.g. *F*, *t*, *r*) with confidence intervals, effect sizes, degrees of freedom and *P* value noted<br>*Give P values as exact values whenever suitable.*
☒	☐	For Bayesian analysis, information on the choice of priors and Markov chain Monte Carlo settings
☒	☐	For hierarchical and complex designs, identification of the appropriate level for tests and full reporting of outcomes
☐	☒	Estimates of effect sizes (e.g. Cohen's *d*, Pearson's *r*), indicating how they were calculated

Our web collection on statistics for biologists contains articles on many of the points above.

Software and code

Policy information about availability of computer code

Data collection	The cDNA Libraries were prepared with the Chromium Single Cell 3´ Reagent Kit v2 and v3 and sequenced on an Illumina NovaSeq 6000 (Illumina) sequencer. Reads were aligned to the human reference genome GRCh38-3.0.0 and libraries were demultiplexed and aligned with the 10X Genomics pipeline CellRanger (version 3.0.2). Loom files were generated for each sample by running Velocyto (0.17.17).<br>Tissues sections, analyzed by Spatial Transcriptomics, were stained with Hematoxylin and Eosin Y and imaged with a Zeiss Imager.Z2 Microscope (Carl Zeiss Microscopy GmbH), using the Metafer5 software (version 3.14.3) (MetaSystems Hard & Software GmbH).<br><br>HybISS data were collected with a Zeiss Axio Imager.Z2 epifluorescence microscope (Carl Zeiss Microscopy, GmbH), equipped with a Zeiss Plan-Apochromat 20x/0.8 objective (Carl Zeiss Microscopy, GmbH, 420650-9901), an automatic multi-slide stage (PILine, M-686K011), a Lumencor® SPECTRA X light engine LED source (Lumencor, Inc.) and the quad band Chroma 89402 (DAPI, Cy3, Cy5), the quad band Chroma 89403 (AlexaFluor750), and the single band Zeiss 38HE (AlexaFluor488). Images were obtained with an ORCA-Flash4.0 LT Plus sCMOS camera (2048 × 2048, 16-bit, Hamamatsu Photonics K. K.), using the Zen Blue 2.5 software (Carl Zeiss Microscopy, GmbH).<br><br>SCRINSHOT data were collected with a Zeiss Axio Observer Z.2 fluorescent microscope (Carl Zeiss Microscopy, GmbH) with a Colibri 7 led light source (Carl Zeiss Microscopy, GmbH, 423052-9770-000), equipped with a Zeiss 20X/0.75 Plan-Apochromat, a Zeiss AxioCam 506 Mono digital camera, an automated stage and the Chroma filters: 49000 (DAPI), 49003 (FITC), 49304 (Cy3), 49307 (Cy5), 49310 (Texas Red) and 49007 (Atto740), using the Zen Blue 2.5 software (Carl Zeiss Microscopy GmbH).<br><br>Immunofluorescence image datasets were generated with a Zeiss LSM800 confocal microscope, equipped with a Plan-Apochromat 40X/1.30 oil objective, using the Zen Blue 2.5 software (Carl Zeiss Microscopy GmbH) and a Zeiss LSM780 confocal microscope equipped with a Plan-Apochromat 63X/1.40 oil DIC M27 objective.
Data analysis	For scRNA-Seq analyses, we used the CellRanger 3.0.2 Pipeline (10X Genomics), the Velocyto 0.17.17 and the following R-packages: SeuratDisk 0.0.0.9019, loomR 0.2.1.9000, SeuratWrappers 0.3.0, Seurat 4.0.5, DoubletFinder 2.0.3, sctransform 0.3.2, MAST 1.18.0, slingshot 2.0.0,

tradeSeq 1.6.0, fastcluster 1.2.3, CellChat 1.1.0, nichenetr 1.0.0, igraph 1.2.7 and fpc 2.2-9.
For scVelo analyses, we used the following Python 3.9.7 packages: cellrank 1.5.1, loompy 3.0.6, matplotlib 3.5.1, numpy 1.20.3, pandas 1.4.1, scanpy 1.8.2, scvelo 0.2.4.
For ST analyses, we used the Space Ranger 1.0.0 Pipeline (10X Genomics), the R-packages: harmony_0.1.0 and STutility 0.1.0 and the Python-package: stereoscope v.03.
For HybISS analyses, we used an in-house pipeline based on MATLAB (https://github.com/Moldia/iss_starfish). Cell type assignment was done with the Probabilistic cell-typing for in situ sequencing (PciSeq) function implemented in Matisse (1.1.0) (https://github.com/Moldia/Matisse).
For Tangram cell typing, we used the package tangram-sc (1.0.2) in Python 3.8.5.
For SCRINSHOT analyses, Zen Blue 2.5 (Carl Zeiss Microscopy GmbH), ImageJ Fiji, R 4.1.0, R-Studio 1.4.1106 and CellProfiler 4.13.
For neuroendocrine cell spatial analyses, we used the following Python 3.7.12 packages: anndata 0.7.8, scanpy 1.8.2, leidenalg 0.8.8, matplotlib 3.5.1, numpy 1.21.5, pandas 1.3.5.
The browser-based representation of the data is available with the TissUUmaps tool (https://tissuumaps.github.io/).

The scripts for all analyses can be accessed in 10.5281/zenodo.7143091.

For manuscripts utilizing custom algorithms or software that are central to the research but not yet described in published literature, software must be made available to editors and reviewers. We strongly encourage code deposition in a community repository (e.g. GitHub). See the Nature Portfolio guidelines for submitting code & software for further information.

Data

Policy information about availability of data

All manuscripts must include a data availability statement. This statement should provide the following information, where applicable:

- Accession codes, unique identifiers, or web links for publicly available datasets
- A description of any restrictions on data availability
- For clinical datasets or third party data, please ensure that the statement adheres to our policy

The GRCh38-3.0.0 reference human genome can be accessed from 10X Genomics, in the section: References - 3.0.0 (November 19, 2018)
All sequencing data have been deposited in GEO (GSE215898), comprising of single cell data (GSE215895) and spatial transcriptomics data (GSE215897).
The datasets generated during and/or analyzed during the current study are available at https://hdca-sweden.scilifelab.se/tissues-overview/lung/.
The scRNA-Seq data can be additionally accessed in https://cells.ucsc.edu/?ds=lung-dev. scRNA-Seq datasets of individual-donors can be accessed at DOI: 10.5281/zenodo.6386452.
The used scRNA-Seq datasets, containing subsets of the whole-dataset and of the mesenchymal cell dataset are available at 10.5281/zenodo.7143999.
The raw-data of the fluorescence images can be accessed at DOI: 10.1101/2022.01.11.475631 and DOI: 10.5281/zenodo.6673650.
Spatial Transcriptomics (ST) raw data can be accessed at DOI: 10.5281/zenodo.6661019.
scVelo datasets and analysis files can be accessed at DOI: 10.5281/zenodo.6673667.
Raw-image datasets of HybISS (180 GB) and SCRINSHOT (683 GB) are available from the corresponding authors on reasonable request because of data size limitations.

Field-specific reporting

Please select the one below that is the best fit for your research. If you are not sure, read the appropriate sections before making your selection.

☒ Life sciences ☐ Behavioural & social sciences ☐ Ecological, evolutionary & environmental sciences

For a reference copy of the document with all sections, see nature.com/documents/nr-reporting-summary-flat.pdf

Life sciences study design

All studies must disclose on these points even when the disclosure is negative.

Sample size	No statistical method was used to predetermine sample size.
Data exclusions	Low quality scRNA-Seq cDNA libraries based on the number of detected genes and the percent of mitochondrial genes in addition to potential multiplets were excluded from further analyses. Regarding all other methods no data were excluded from the analyses.
Replication	All attempts at replication were successful.
Randomization	The experiments were not randomized.
Blinding	The Investigators were not blinded to allocation during experiments and outcome assessment.

Behavioural & social sciences study design

All studies must disclose on these points even when the disclosure is negative.

Study description	*Briefly describe the study type including whether data are quantitative, qualitative, or mixed-methods (e.g. qualitative cross-sectional, quantitative experimental, mixed-methods case study).*

Research sample	*State the research sample (e.g. Harvard university undergraduates, villagers in rural India) and provide relevant demographic information (e.g. age, sex) and indicate whether the sample is representative. Provide a rationale for the study sample chosen. For studies involving existing datasets, please describe the dataset and source.*
Sampling strategy	*Describe the sampling procedure (e.g. random, snowball, stratified, convenience). Describe the statistical methods that were used to predetermine sample size OR if no sample-size calculation was performed, describe how sample sizes were chosen and provide a rationale for why these sample sizes are sufficient. For qualitative data, please indicate whether data saturation was considered, and what criteria were used to decide that no further sampling was needed.*
Data collection	*Provide details about the data collection procedure, including the instruments or devices used to record the data (e.g. pen and paper, computer, eye tracker, video or audio equipment) whether anyone was present besides the participant(s) and the researcher, and whether the researcher was blind to experimental condition and/or the study hypothesis during data collection.*
Timing	*Indicate the start and stop dates of data collection. If there is a gap between collection periods, state the dates for each sample cohort.*
Data exclusions	*If no data were excluded from the analyses, state so OR if data were excluded, provide the exact number of exclusions and the rationale behind them, indicating whether exclusion criteria were pre-established.*
Non-participation	*State how many participants dropped out/declined participation and the reason(s) given OR provide response rate OR state that no participants dropped out/declined participation.*
Randomization	*If participants were not allocated into experimental groups, state so OR describe how participants were allocated to groups, and if allocation was not random, describe how covariates were controlled.*

Ecological, evolutionary & environmental sciences study design

All studies must disclose on these points even when the disclosure is negative.

Study description	*Briefly describe the study. For quantitative data include treatment factors and interactions, design structure (e.g. factorial, nested, hierarchical), nature and number of experimental units and replicates.*
Research sample	*Describe the research sample (e.g. a group of tagged Passer domesticus, all Stenocereus thurberi within Organ Pipe Cactus National Monument), and provide a rationale for the sample choice. When relevant, describe the organism taxa, source, sex, age range and any manipulations. State what population the sample is meant to represent when applicable. For studies involving existing datasets, describe the data and its source.*
Sampling strategy	*Note the sampling procedure. Describe the statistical methods that were used to predetermine sample size OR if no sample-size calculation was performed, describe how sample sizes were chosen and provide a rationale for why these sample sizes are sufficient.*
Data collection	*Describe the data collection procedure, including who recorded the data and how.*
Timing and spatial scale	*Indicate the start and stop dates of data collection, noting the frequency and periodicity of sampling and providing a rationale for these choices. If there is a gap between collection periods, state the dates for each sample cohort. Specify the spatial scale from which the data are taken*
Data exclusions	*If no data were excluded from the analyses, state so OR if data were excluded, describe the exclusions and the rationale behind them, indicating whether exclusion criteria were pre-established.*
Reproducibility	*Describe the measures taken to verify the reproducibility of experimental findings. For each experiment, note whether any attempts to repeat the experiment failed OR state that all attempts to repeat the experiment were successful.*
Randomization	*Describe how samples/organisms/participants were allocated into groups. If allocation was not random, describe how covariates were controlled. If this is not relevant to your study, explain why.*
Blinding	*Describe the extent of blinding used during data acquisition and analysis. If blinding was not possible, describe why OR explain why blinding was not relevant to your study.*

Did the study involve field work? ☐ Yes ☒ No

Reporting for specific materials, systems and methods

We require information from authors about some types of materials, experimental systems and methods used in many studies. Here, indicate whether each material, system or method listed is relevant to your study. If you are not sure if a list item applies to your research, read the appropriate section before selecting a response.

Materials & experimental systems

n/a	Involved in the study
☐	☒ Antibodies
☒	☐ Eukaryotic cell lines
☒	☐ Palaeontology and archaeology
☒	☐ Animals and other organisms
☐	☒ Human research participants
☒	☐ Clinical data
☒	☐ Dual use research of concern

Methods

n/a	Involved in the study
☒	☐ ChIP-seq
☒	☐ Flow cytometry
☒	☐ MRI-based neuroimaging

Antibodies

Antibodies used

Primary antibodies:
anti-PHOX2B goat polyclonal antibody (R&D Systems, AF4940-SP, working dilution (wd:2µg/mL, Lot No: CBDC0321061)
anti-DLL3 rabbit monoclonal antibody (Cell Signalling Technology, 71804, clone: E3J5R, wd:1:50, Lot No: 3)
anti-NF-M mouse monoclonal antibody (DSHB, 2H3, clone: 2H3, wd:2µg/ml, Lot No: 7/16/20)
anti-COL13A1 rabbit polyclonal antibody (NovusBio, NBP2-13854, wd:1:100, Lot No: 21613)
Cy3 anti-αSmooth Muscle Actin (ACTA2) mouse monoclonal antibody (Sigma Aldrich, C6198, clone:1A4, wd:1:2000, Lot No: 124M4815V)
anti-GHRL rat monoclonal antibody (R&D Systems, MAB8200-SP, clone: 883622, wd:1.25µg/ml, Lot No: CILU0220021)
anti-GRP rabbit polyclonal antibody (Bioss, bs-0011R, wd:1:200, Lot No: AI08112480)
anti-SOX10 goat polyclonal antibody (R&D Systems, AF2864-SP, wd:5µg/ml, Lot No: VRY1220031)
anti-MASH1 rabbit monoclonal antibody (Abcam, ab240385, clone: EPR19840, wd:1:100, Lot No: GR3258827-2)
anti-ISL1 mouse monoclonal antibody (DSHB, 40.2D6, clone: 40.2D6, wd:1.3µg/ml, Lot No: 2/18/21)
anti-E-cadherin mouse Alexa Flour 555 (BD Biosciences, 560064, clone: 36/E-Cadherin, wd:1:100, Lot No: 4337645),
anti-E-cadherin mouse Alexa Flour 647 (BD Biosciences, 560062, clone: 36/E-Cadherin, wd:1:100, Lot No: 7040557)
anti-p63α rabbit monoclonal antibody (Cell Signaling Technologies, 13109, clone: D2K8X, wd:1:100, Lot No: 3)
anti-Krt5 chicken polyclonal antibody (Biolegend, 905901, wd:1:400, Lot No: B29722)
anti-E-cadherin rat monoclonal antibody (ThermoFisher Scientific, 13-1900, clone: ECCD-2, wd:10µg/ml, Lot SL2474201)
anti-KI67 rabbit polyclonal antibody (Invitrogen, PA5-19462, wd:1:500, Lot:GR32000471)
anti-KRT17 rabbit polyclonal antibody (Sigma Aldrich, HPA000452, wd:1:200, Lot:A08686)
anti-SST mouse monoclonal antibody (NovusBio, NBP2-37447, clone: 7G5, wd:1:200, Lot:160825)
and anti-SSTR2 rabbit polyclonal antibody (Proteintech, 20404-1-AP, wd:1:100, Lot:00013177)
Secondary antibodies:
Alexa Fluor 488 donkey anti-goat (Jackson ImmunoResearch, 705-545-147, wd: 1:400)
Cy3 donkey anti-rabbit (Jackson ImmunoResearch, 711-165-152, wd: 1:400)
Cy5 donkey anti-mouse (Jackson ImmunoResearch, 715-175-151, wd: 1:400)
Cy5 donkey anti-rabbit (Jackson ImmunoResearch, 711-175-152, wd: 1:400)
Alexa Fluor 488 donkey anti-rat (Jackson ImmunoResearch, 712-545-153, wd:1:400)
Alexa Fluor 488 donkey anti-rabbit (ThermoFischer Scientific, A-21206, wd:1:400)
Alexa Fluor Cy3 donkey anti-chicken (Jackson ImmunoResearch, 703-165-155, wd:1:400)
Alexa Fluor 647 goat anti-mouse (Invitrogen, A32728, wd:1:250)
Alexa Fluor 488 goat anti-rabbit (Invitrogen, A11034, wd:1:250)
Alexa Fluor 790 donkey anti-rabbit (Jackson ImmunoResearch, 711-656-152, wd:1:400)
Alexa Fluor Cy3 donkey anti-mouse (Jackson ImmunoResearch, 715-165-151, wd:1:400)
Alexa Fluor 647 donkey anti-rabbit (Jackson ImmunoResearch, 711-606-152, wd:1:400)

Validation

anti-PHOX2B goat polyclonal antibody (R&D Systems, AF4940-SP): Manufacturer provides information regarding the species-reactivity and citation(s) for usage in tissue sections for immunohistochemistry/immunofluorescence.
anti-DLL3 rabbit monoclonal antibody (Cell Signalling Technology, 71804): Manufacturer provides information and citation(s) regarding the species-reactivity and usage on tissue sections for immunohistochemistry/immunofluorescence.
anti-NF-M mouse monoclonal antibody (DSHB, 2H3): Manufacturer provides information regarding the species-reactivity and usage on tissue sections for immunohistochemistry/immunofluorescence.
anti-COL13A1 rabbit polyclonal antibody (NovusBio, NBP2-13854): Manufacturer provides information regarding the species-reactivity and usage on tissue sections for immunohistochemistry/immunofluorescence.
Cy3 anti-αSmooth Muscle Actin (ACTA2) mouse monoclonal antibody (Sigma Aldrich, C6198): Manufacturer provides information and citation(s) regarding the species-reactivity and usage on tissue sections for immunohistochemistry/immunofluorescence.
anti-GHRL rat monoclonal antibody (R&D Systems, MAB8200-SP): Manufacturer provides information and citation(s) regarding the species-reactivity and usage on tissue sections for immunohistochemistry/immunofluorescence.
anti-GRP rabbit polyclonal antibody (Bioss, bs-0011R): Manufacturer provides information regarding the species-reactivity and usage on tissue sections for immunohistochemistry.
anti-SOX10 goat polyclonal antibody (R&D Systems, AF2864-SP): Manufacturer provides information and citation(s) regarding the species-reactivity and usage on tissue sections for immunohistochemistry/immunofluorescence.
anti-MASH1 rabbit monoclonal antibody (Abcam, ab240385): Manufacturer provides information regarding the species-reactivity and usage on tissue sections for immunohistochemistry/immunofluorescence.
anti-ISL1 mouse monoclonal antibody (DSHB, 40.2D6): Manufacturer provides information regarding the species-reactivity and usage on tissue sections for immunohistochemistry/immunofluorescence.
anti-E-cadherin mouse Alexa Flour 555 (BD Biosciences, 560064): Manufacturer provides information and citation(s) regarding the species-reactivity and usage on tissue sections for immunohistochemistry/immunofluorescence.
anti-E-cadherin mouse Alexa Flour 647 (BD Biosciences, 560062): Manufacturer provides information and citation(s) regarding the

species-reactivity and usage on tissue sections for immunohistochemistry/immunofluorescence.
anti-p63α rabbit monoclonal (Cell Signalling Technologies, 13109): Manufacturer provides information and citation(s) regarding the species-reactivity and usage for immunofluorescence.
anti-Krt5 chicken polyclonal (Biolegend, 905901). Manufacturer provides information and citation(s) regarding the species-reactivity and usage on tissue sections for immunohistochemistry/immunofluorescence.
anti-E-cadherin rat monoclonal antibody (ThermoFisher Scientific, 13-1900). Manufacturer provides information and citation(s) regarding the species-reactivity and usage on tissue sections for immunohistochemistry/immunofluorescence
anti-KI67 rabbit polyclonal antibody (Invitrogen, PA5-19462). Manufacturer provides information and citation(s) regarding the species-reactivity and usage on tissue sections for immunohistochemistry/immunofluorescence
anti-KRT17 rabbit polyclonal antibody (Sigma Aldrich, HPA000452). Manufacturer provides information and citation(s) regarding the species-reactivity and usage on tissue sections for immunohistochemistry/immunofluorescence
anti-SST mouse monoclonal antibody (NovusBio, NBP2-37447). Manufacturer provides information and citation(s) regarding the species-reactivity and usage on tissue sections for immunohistochemistry/immunofluorescence.
and anti-SSTR2 rabbit polyclonal antibody (Proteintech, 20404-1-AP).Manufacturer provides information and citation(s) regarding the species-reactivity and usage on tissue sections for immunohistochemistry/immunofluorescence.

Eukaryotic cell lines

Policy information about cell lines

Cell line source(s) | *State the source of each cell line used.*

Authentication | *Describe the authentication procedures for each cell line used OR declare that none of the cell lines used were authenticated.*

Mycoplasma contamination | *Confirm that all cell lines tested negative for mycoplasma contamination OR describe the results of the testing for mycoplasma contamination OR declare that the cell lines were not tested for mycoplasma contamination.*

Commonly misidentified lines (See ICLAC register) | *Name any commonly misidentified cell lines used in the study and provide a rationale for their use.*

Palaeontology and Archaeology

Specimen provenance | *Provide provenance information for specimens and describe permits that were obtained for the work (including the name of the issuing authority, the date of issue, and any identifying information). Permits should encompass collection and, where applicable, export.*

Specimen deposition | *Indicate where the specimens have been deposited to permit free access by other researchers.*

Dating methods | *If new dates are provided, describe how they were obtained (e.g. collection, storage, sample pretreatment and measurement), where they were obtained (i.e. lab name), the calibration program and the protocol for quality assurance OR state that no new dates are provided.*

☐ Tick this box to confirm that the raw and calibrated dates are available in the paper or in Supplementary Information.

Ethics oversight | *Identify the organization(s) that approved or provided guidance on the study protocol, OR state that no ethical approval or guidance was required and explain why not.*

Note that full information on the approval of the study protocol must also be provided in the manuscript.

Animals and other organisms

Policy information about studies involving animals; ARRIVE guidelines recommended for reporting animal research

Laboratory animals | *For laboratory animals, report species, strain, sex and age OR state that the study did not involve laboratory animals.*

Wild animals | *Provide details on animals observed in or captured in the field; report species, sex and age where possible. Describe how animals were caught and transported and what happened to captive animals after the study (if killed, explain why and describe method; if released, say where and when) OR state that the study did not involve wild animals.*

Field-collected samples | *For laboratory work with field-collected samples, describe all relevant parameters such as housing, maintenance, temperature, photoperiod and end-of-experiment protocol OR state that the study did not involve samples collected from the field.*

Ethics oversight | *Identify the organization(s) that approved or provided guidance on the study protocol, OR state that no ethical approval or guidance was required and explain why not.*

Note that full information on the approval of the study protocol must also be provided in the manuscript.

Human research participants

Policy information about studies involving human research participants

Population characteristics | Tissue donors included pregnant women 18 years of age or older, fluent in Swedish that wanted to terminate their pregnancy. Exclusion criteria related to the abortions performed for any medical reasons, by socially compromised women

and/or by women showing any signs that the consent may not be informed. The 17 lung samples were retrieved from fetuses between 5- and 14-weeks post conception (5 pcw: 1 sample, 5.5 pcw: 1 sample, 6 pcw: 2 samples, 7 pcw: 2 samples, 8 pcw: 1 sample, 8.5 pcw: 2 samples, 10 pcw: 1 sample, 11.5 pcw: 2 samples, 12 pcw: 2 samples, 13 pcw: 2 sample, 14 pcw: 1 sample). The gender was determined bioinformatically, after the tissue analysis showing that the tissues were collected from 10 female and 7 male fetuses. Thus there was no bias.

Recruitment

The tissue donors were recruited among pregnant women after their decision to terminate their pregnancy. The referral to hospitals was done by a central office for all abortion clinics in the Stockholm region and according to our information it was random. The recruitments were done by midwifes that were not involved in the conducted research. Thus, there was no bias regarding which women were recruited. Inclusion criteria: 18 years of age or older, fluent in Swedish. Exclusion criterias: Abortions performed for any medical reasons, by socially compromised women and/or by women showing any signs that the consent may not be informed. There was not any type of compensation to the tissue donors.

Ethics oversight

The use of human fetal material from the elective routine abortions was approved by the Swedish National Board of Health and Welfare and the analysis using this material was approved by the Swedish Ethical Review Authority (2018/769-31). The clinical staff acquired the informed written consent by the donors, that the retrieved material can be used for research purposes and about their ability to withdraw their consent anytime. Then, the fetal material was transferred to the research prenatal material.

Note that full information on the approval of the study protocol must also be provided in the manuscript.

ChIP-seq

Data deposition

- [ ] Confirm that both raw and final processed data have been deposited in a public database such as GEO.
- [ ] Confirm that you have deposited or provided access to graph files (e.g. BED files) for the called peaks.

Data access links
May remain private before publication.

For "Initial submission" or "Revised version" documents, provide reviewer access links. For your "Final submission" document, provide a link to the deposited data.

Files in database submission

Provide a list of all files available in the database submission.

Genome browser session
(e.g. UCSC)

Provide a link to an anonymized genome browser session for "Initial submission" and "Revised version" documents only, to enable peer review. Write "no longer applicable" for "Final submission" documents.

Methodology

Replicates

Describe the experimental replicates, specifying number, type and replicate agreement.

Sequencing depth

Describe the sequencing depth for each experiment, providing the total number of reads, uniquely mapped reads, length of reads and whether they were paired- or single-end.

Antibodies

Describe the antibodies used for the ChIP-seq experiments; as applicable, provide supplier name, catalog number, clone name, and lot number.

Peak calling parameters

Specify the command line program and parameters used for read mapping and peak calling, including the ChIP, control and index files used.

Data quality

Describe the methods used to ensure data quality in full detail, including how many peaks are at FDR 5% and above 5-fold enrichment.

Software

Describe the software used to collect and analyze the ChIP-seq data. For custom code that has been deposited into a community repository, provide accession details.

Flow Cytometry

Plots

Confirm that:

- [ ] The axis labels state the marker and fluorochrome used (e.g. CD4-FITC).
- [ ] The axis scales are clearly visible. Include numbers along axes only for bottom left plot of group (a 'group' is an analysis of identical markers).
- [ ] All plots are contour plots with outliers or pseudocolor plots.
- [ ] A numerical value for number of cells or percentage (with statistics) is provided.

Methodology

Sample preparation

Describe the sample preparation, detailing the biological source of the cells and any tissue processing steps used.

Instrument | *Identify the instrument used for data collection, specifying make and model number.*

Software | *Describe the software used to collect and analyze the flow cytometry data. For custom code that has been deposited into a community repository, provide accession details.*

Cell population abundance | *Describe the abundance of the relevant cell populations within post-sort fractions, providing details on the purity of the samples and how it was determined.*

Gating strategy | *Describe the gating strategy used for all relevant experiments, specifying the preliminary FSC/SSC gates of the starting cell population, indicating where boundaries between "positive" and "negative" staining cell populations are defined.*

☐ Tick this box to confirm that a figure exemplifying the gating strategy is provided in the Supplementary Information.

Magnetic resonance imaging

Experimental design

Design type | *Indicate task or resting state; event-related or block design.*

Design specifications | *Specify the number of blocks, trials or experimental units per session and/or subject, and specify the length of each trial or block (if trials are blocked) and interval between trials.*

Behavioral performance measures | *State number and/or type of variables recorded (e.g. correct button press, response time) and what statistics were used to establish that the subjects were performing the task as expected (e.g. mean, range, and/or standard deviation across subjects).*

Acquisition

Imaging type(s) | *Specify: functional, structural, diffusion, perfusion.*

Field strength | *Specify in Tesla*

Sequence & imaging parameters | *Specify the pulse sequence type (gradient echo, spin echo, etc.), imaging type (EPI, spiral, etc.), field of view, matrix size, slice thickness, orientation and TE/TR/flip angle.*

Area of acquisition | *State whether a whole brain scan was used OR define the area of acquisition, describing how the region was determined.*

Diffusion MRI ☐ Used ☐ Not used

Preprocessing

Preprocessing software | *Provide detail on software version and revision number and on specific parameters (model/functions, brain extraction, segmentation, smoothing kernel size, etc.).*

Normalization | *If data were normalized/standardized, describe the approach(es): specify linear or non-linear and define image types used for transformation OR indicate that data were not normalized and explain rationale for lack of normalization.*

Normalization template | *Describe the template used for normalization/transformation, specifying subject space or group standardized space (e.g. original Talairach, MNI305, ICBM152) OR indicate that the data were not normalized.*

Noise and artifact removal | *Describe your procedure(s) for artifact and structured noise removal, specifying motion parameters, tissue signals and physiological signals (heart rate, respiration).*

Volume censoring | *Define your software and/or method and criteria for volume censoring, and state the extent of such censoring.*

Statistical modeling & inference

Model type and settings | *Specify type (mass univariate, multivariate, RSA, predictive, etc.) and describe essential details of the model at the first and second levels (e.g. fixed, random or mixed effects; drift or auto-correlation).*

Effect(s) tested | *Define precise effect in terms of the task or stimulus conditions instead of psychological concepts and indicate whether ANOVA or factorial designs were used.*

Specify type of analysis: ☐ Whole brain ☐ ROI-based ☐ Both

Statistic type for inference (See Eklund et al. 2016) | *Specify voxel-wise or cluster-wise and report all relevant parameters for cluster-wise methods.*

Correction | *Describe the type of correction and how it is obtained for multiple comparisons (e.g. FWE, FDR, permutation or Monte Carlo).*

Models & analysis

n/a	Involved in the study
☐	☐ Functional and/or effective connectivity
☐	☐ Graph analysis
☐	☐ Multivariate modeling or predictive analysis

Functional and/or effective connectivity	*Report the measures of dependence used and the model details (e.g. Pearson correlation, partial correlation, mutual information).*
Graph analysis	*Report the dependent variable and connectivity measure, specifying weighted graph or binarized graph, subject- or group-level, and the global and/or node summaries used (e.g. clustering coefficient, efficiency, etc.).*
Multivariate modeling and predictive analysis	*Specify independent variables, features extraction and dimension reduction, model, training and evaluation metrics.*
